# Fabrication of a Metal Micro Mold by Using Pulse Micro Electroforming

**DOI:** 10.3390/mi9050203

**Published:** 2018-04-27

**Authors:** Xiaolei Chen, Li Liu, Junfeng He, Fei Zuo, Zhongning Guo

**Affiliations:** 1 School of Electro-mechanical Engineering, Guangdong University of Technology, Guangzhou 510016, China; xlchen@gdut.edu.cn (X.C.); llgdut@163.com (L.L.); feizuo108@outlook.com (F.Z.); znguo@gdut.edu.cn (Z.G.); 2 Guangzhou Key Laboratory of Nontraditional Machining and Equipment, Guangzhou 510006, China

**Keywords:** microfluidic devices, micro mold, electroforming, surface quality, surface roughness

## Abstract

Microfluidic devices have been widely used for biomedical and biochemical applications. Due to its unique characteristics, polymethyl methacrylate (PMMA) show great potential in fabricating microfluidic devices. Hot embossing technology has established itself as a popular method of preparing polymer microfluidic devices in both academia and industry. However, the fabrication of the mold used in hot embossing is time-consuming in general and often impractical for economically efficient prototyping. This paper proposes a modified technology for preparing metal micro molds by using pulse micro electroforming directly on metallic substrate, which could save time and reduce costs. In this method, an additive was used to avoid surface defect on deposited nickel. A chemical etching process was performed on the metallic substrate before the electroforming process in order to improve the bonding strength between the deposited structure and substrate. Finally, with the aim of obtaining a metal micro mold with high surface quality (low surface roughness), an orthogonal experiment was designed and conducted to optimize the electroforming parameters. Additionally, metal micro molds with different structures were well prepared by using the optimized parameters.

## 1. Introduction

Microfluidics has been developing for three decades [1], and there is significant development in microfluidic devices for biomedical and biochemical applications, which are used to perform mixing, chemical reactions, particle detection and separation, and so on [2,3,4]. In the early years of microfluidics, devices were predominantly made of materials like glass, quartz or silicon [5]. Recently, compared with silicon and glass, polymethyl methacrylate (PMMA) has attracted more attention for its application in chemical and biological microfluidic applications, due to the advantages of low cost, favorable optical properties, resistance to chemicals, and reduced contamination effects Also, thermoplastics possess inherent robustness to mechanical deformation [6,7].

A number of approaches are available for preparing polymer microfluidic devices, such as micro milling [8], laser ablation [2], etching [9] and lithographie, galvanoformung, abformung (LIGA) [10]. Compared with other methods, hot embossing technology has established itself as a popular method for high-volume fabrication of microfluidic device in both academia and industry [11,12]. This technology involves a comparatively simple process that simplifies the selection of processing parameters, has relatively low requirements for mold structures, has a large range of suitable materials and good availability of commercial products [13].

The functionality and reliability of microfluidic devices are mainly determined by the geometrical tolerances in the channel geometry and surface roughness. It has been reported that devices fabricated by direct writing methods such as micro milling or laser ablation always have poor surface quality [14,15]. In hot embossing processes, all the properties of the replica are directly impacted by the geometric tolerances and the surface quality of the reverse mold structures. Hence, it is important to improve the quality of mold structure.

Generally, the mold structure is fabricated from photolithographic procedures such as silicon bulk micromachining or surface micromachining employing high-aspect-ratio resists such as SU-8 [16]. In the typical conventional procedure for preparing the mold, a silicon or SU-8 photoresist structured primary mold is treated with a conductive layer on the surface, and then electroforming is performed in a galvanic bath to form the secondary mold. As the whole mold, including the structure and substrate needs to be prepared by an electroforming process, the manufacturing of micro structured metal masters is a complex and rather lengthy process, often making it impractical for economically efficient prototyping [17]. Although enhanced current densities could increase the growth speed [18], it also tends to increase the internal stress, which can lead to warped metal mold exhibiting insufficient surface quality. Hence, the electroplated mold needs to be post-processed by mechanical machining. To reduce the time for the testing of novel microfluidic designs and limit the risk of the electroplating process, an alternative technique for generating a high-quality metal mold with a sufficient lifetime for a small-lot replication is required.

This paper proposed a modified method for preparing metal micro molds by using pulse micro electroforming directly on metallic substrate, which could save time and reduce the cost. In this process, an additive was used to avoid surface defects on deposited nickel. A chemical etching process was performed on the metallic substrate before the electroforming process in order to enhance the bonding strength between the deposited structure and substrate. Finally, to obtain a metal micro mold with high surface quality (low surface roughness), an orthogonal experiment was designed and conducted to optimize the electroforming parameters. Also, metal micro molds with different structures were prepared by using the optimized parameters.

## 2. Materials and Methods

### 2.1. Materials and Equipment

In this paper, SU-8 2050 negative photoresist and propylene glycol methyl ether acetate (PGMEA, 1-methoxy-2-propanol acetate) developer (MicroChem Corp., Westborough, MA, USA) were used to prepare the micro electroforming mold. Polished 1 mm thick stainless steel (1Cr18Ni9Ti) sheet was employed as the substrate. An n-methyl pyrrolidinone solution was used to remove the cross-linked SU-8 structure after micro electroforming. The structures and roughness were examined using a confocal laser scanning microscope (CSLM, Olympus LEXT OLS4000, Olympus Corporation, Tokyo, Japan).

### 2.2. Methods

#### 2.2.1. Preparation of the SU-8 Mold

**Figure 1:**
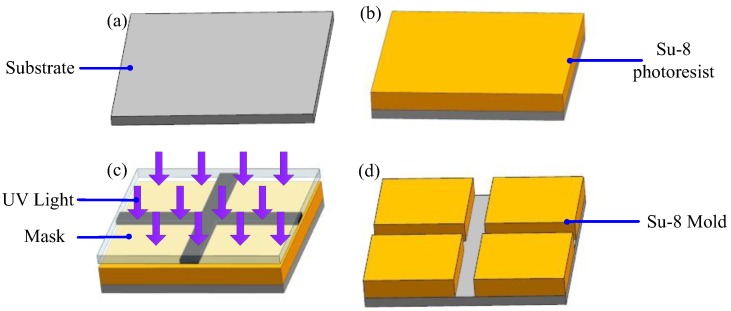
The schematic diagram of process for preparing the SU-8 mold. (**a**) Pretreatment of the substrate; (**b**) Photoresist coating and softbake; (**c**) Exposure; (**d**) Post-exposure bake and development.

Figure 1 shows the schematic diagram of the process for preparing the SU-8 mold.

(1) Pretreatment of the Substrate

In order to obtain the well adhesion between the substrate and photoresist, the substrate should be pre-treated before the photoresist is coated.

First, the stainless-steel substrate was put into the acetone solution in an ultrasonic cleaner to remove the oil film from the substrate. Second, acid washing was employed to remove the oxide film from the substrate surface. Third, the substrate was put into the acetone solution again to remove any remaining acid. Finally, the substrate was rinsed with deionized water and placed into a drying oven with a temperature of 150 °C for 10 min to remove the residual water (Figure 1a).

(2) Photoresist Coating and Softbake

The photoresist was spin coated onto the substrate with a spin speed of 2000 rpm and a photoresist layer with a thickness of 110 ± 10 μm was obtained, which was then placed on a hotplate. The temperature of the hotplate was increased from room temperature to 65 °C, kept for 10 min, and then increased to 95 °C maintained for 150 min. Last, the substrate was cooled to room temperature (Figure 1b).

(3) Exposure, Post-Exposure Bake and Development

**Figure 2:**
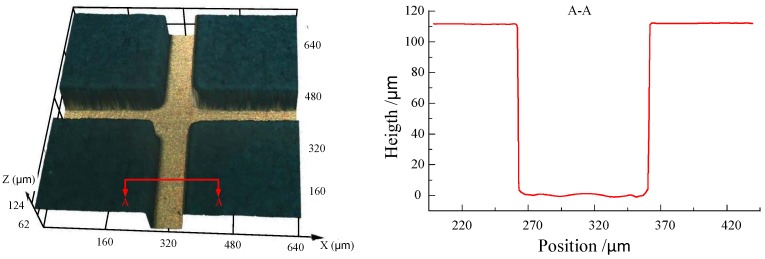
SU-8 molds with a crossing structure.

The ultraviolet (UV) exposure was done by using a mask aligner with wave energy of 365 nm and 6 mW·cm^−2^ (Figure 1c). As the thickness of the SU-8 photoresist was 110 ± 10 μm, the UV dosage was about 240 mJ·cm^−2^. Then the post-exposure bake process was performed on a hotplate: the temperature was kept at 65 °C for 10 min and then at 95 °C for 30 min. After the substrate was cooled to room temperature, the photoresist was developed by using pure PGMEA with gentle ultrasonic agitation for 10 min until the development was complete. After the development, the substrate was rinsed with deionized water to remove the residual pure PGMEA. Last, the substrate was baked at 50 °C for 10 min to remove any residual water, and the SU-8 mold with structure was prepared (Figure 1d). Figure 2 shows the SU-8 mold prepared with a crossing structure.

#### 2.2.2. Micro Electroforming Process

**Figure 3:**
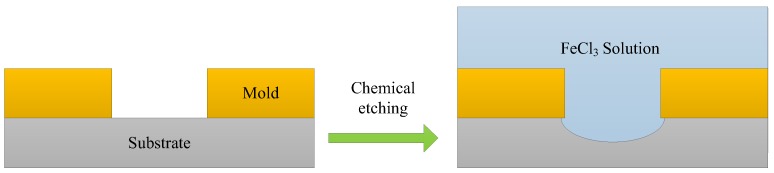
Chemical etching process.

**Figure 4:**
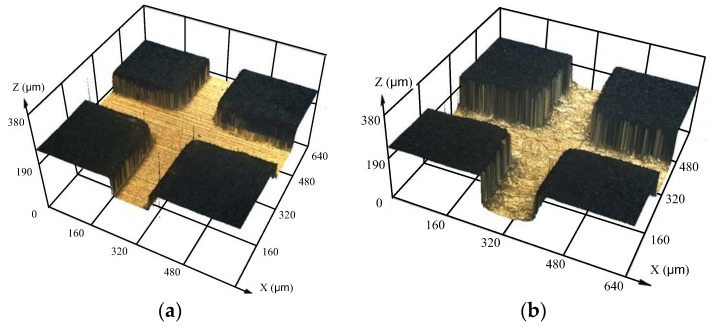
The change in the morphology of the substrate surface. (**a**) Before the chemical etching process; (**b**) After the chemical etching process.

Before micro electroforming, chemical etching process was performed to roughen the substrate as well as increase the contact area between the electroforming deposit and substrate, which was useful for enhancing the bonding strength between the electroforming deposit and the stainless-steel substrate (as shown in Figure 3). In this paper, the chemical etching process was performed in FeCl_3_ solution with a concentration of 150 g/L, temperature of 25 °C, and etching time of 45 s. As the etching time was short, there was no effect on the SU-8 mold. Figure 4 shows the morphology of the substrate surface after chemical etching.

**Figure 5:**
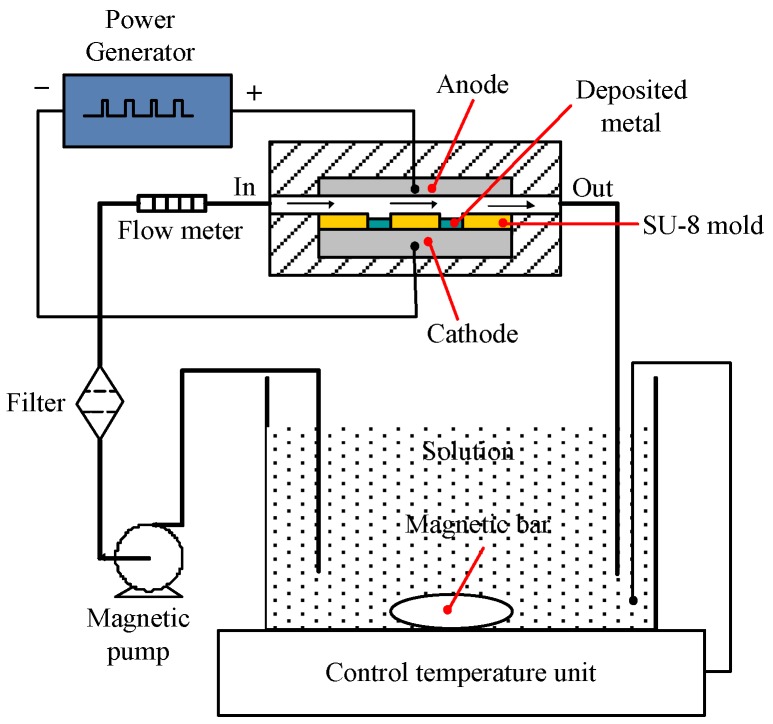
The schematic of the electroforming process.

**Figure 6:**
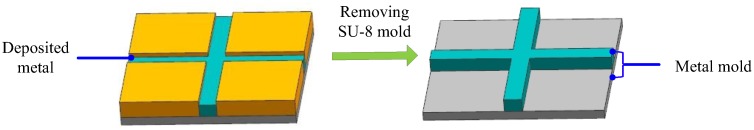
Removal of SU-8 photoresist.

Figure 5 shows the schematic experimental setup of the electroforming process, which consisted of a pulse power generator, an electrolytic cell, a temperature control unit, and a magnetic bar. In this experiment, nickel sheet was used as the anode, and when a sufficient voltage was applied, nickel ions were deposited onto the exposed regions of the stainless-steel substrate with SU-8 mold structure, which acted as a cathode. After the electroforming process, the SU-8 photoresist was removed by putting the substrate in the n-methyl pyrrolidinone solution with ultrasonic-assistance at the frequency of 60 kHz for about 30 min. Then, the metal micro mold was obtained as shown in Figure 6. The electroforming experimental parameters are listed in Table 1.

## 3. Results and Discussion

### 3.1. Effect of Additive on the Surface Quality of the Electroforming Deposit

In the electroforming process, the substrate is connected with a cathode, the nickel ion is deposited on the substrate (Ni^2+^ + 2e→Ni), and the hydrogen bubbles are generated on the substrate at the same time (2H^+^ + 2e→H_2_). The morphology of the deposited surface depends on metallic ions (Ni^2+^) and H^+^ existing on the substrate surface. Due to the low surface tension of the electroforming solution, the buoyancy of the hydrogen bubbles is low, the escape of the hydrogen bubbles is restricted, and most of them adhere on the deposited region. Because the hydrogen bubbles are insulated, they inhibit the metal ion deposition, leading to the pinholes and pits on the deposit surface.

**Figure 7:**
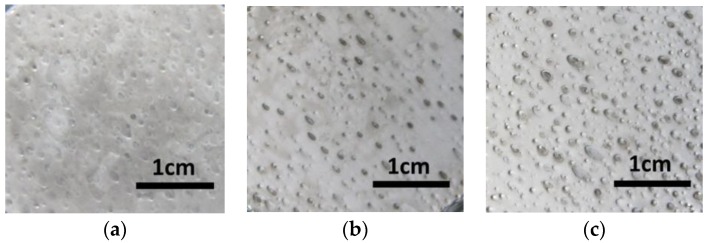
Surface of the deposit with different current density. (**a**) 3 A/dm^2^; (**b**) 5 A/dm^2^; (**c**) 7 A/dm^2^.

**Figure 8:**
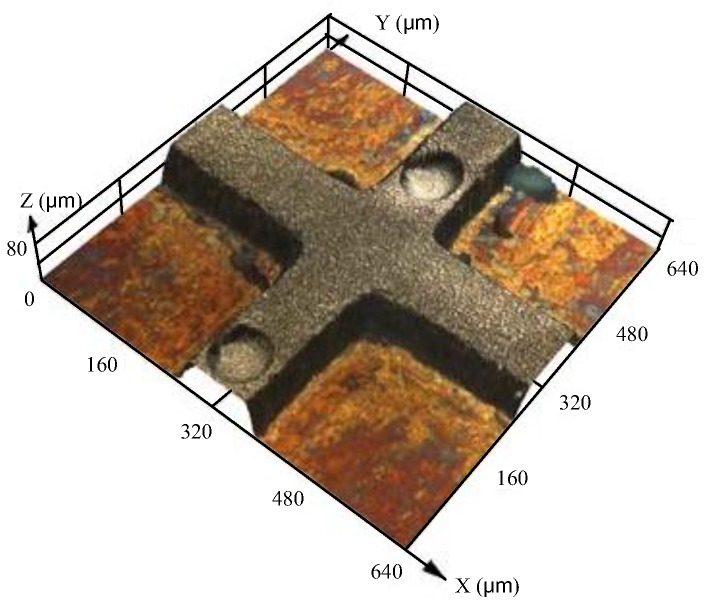
Metal micro mold prepared with current density of 5 A/dm^2^.

Figure 7 shows the surface of the electroforming deposition with different current densities. There were evident pinholes and pits on the surface of the deposition. Also, the defects became more serious with the increasing current density. With the increasing current density, the quantity of hydrogen bubbles increased, due to the adherence of hydrogen bubbles on the deposited surface, the pinholes and pits in the electroforming deposit accumulated and lead to a poor surface. Figure 8 shows the micro mold prepared with the current density of 5 A/dm^2^; there was evident defects on the surface of mold, which would affect its function. Hence, it is important to improve the surface quality of deposits for its application.

**Figure 9:**
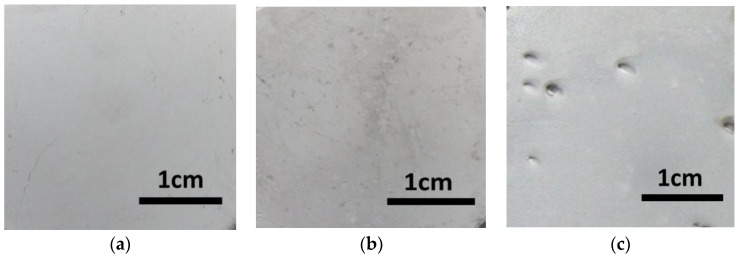
Surface of the deposit with additive. (**a**) 3 A/dm^2^; (**b**) 5 A/dm^2^; (**c**) 7 A/dm^2^.

**Figure 10:**
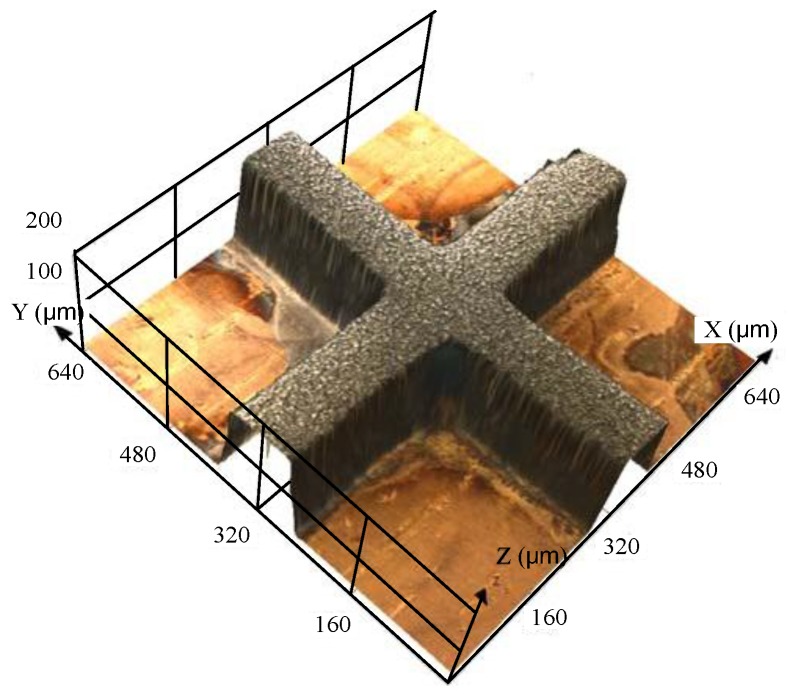
Metal micro mold prepared with additive.

In order to improve the deposit quality, the hydrogen bubbles should be removed from the deposited region as soon as possible. In this experiment, wetting agent (NaC_12_H_25_SO_3_ with the concentration of 0.08 g/L) as an additive was added to the electroforming solution to increase the surface tension of the electroforming solution, which helps the hydrogen bubbles to escape from the deposited surface. As shown in Figure 9, by using the additive, there was no evident defect on the surface of the electroforming deposition with a current density between 3 A/dm^2^ and 5 A/dm^2^. When the current density increased to 7 A/dm^2^, some pits appeared on the surface of deposit. This indicated that with the additive, the hydrogen bubbles could escape from the electroforming region smoothly at a low current density, and it was useful for improving the surface quality. With the current density increased, the deposition rate was accelerated and the quantity of hydrogen bubbles were increased at the same time; some hydrogen bubbles could not escape from the electroforming region leading to the defects on the surface of deposit. Hence, additive was a useful means for improving the deposited surface with low current density (less than 7 A/dm^2^). Figure 10 shows a metal micro mold prepared with additive, compared to that with no additive, there was no evident pits on the deposited surface, and the surface quality was improved significantly. Therefore, NaC_12_H_25_SO_3_ with a concentration of 0.08 g/L was added to the electroforming solution in the experiment.

### 3.2. Effect of Chemical Etching on the Bonding Strength

**Figure 11:**
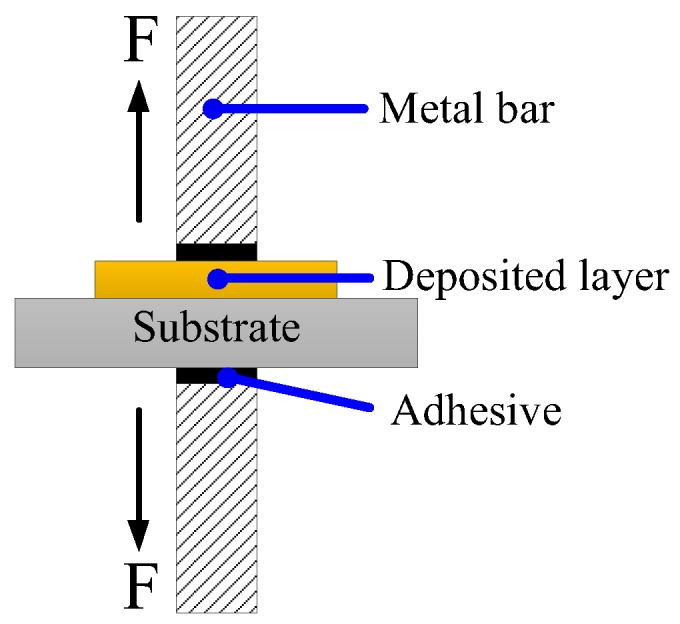
The schematic of testing bonding strength.

This paper proposed a pretreatment process with chemical etching to enhance the bonding strength between the deposit and the substrate. In this section, a tensile test was performed to investigate the bonding strength. As shown in Figure 11, the electroforming deposit and the substrate were connected with a metal bar by using adhesive, and a pull force was applied on both metal bars to test the bonding strength.

**Figure 12:**
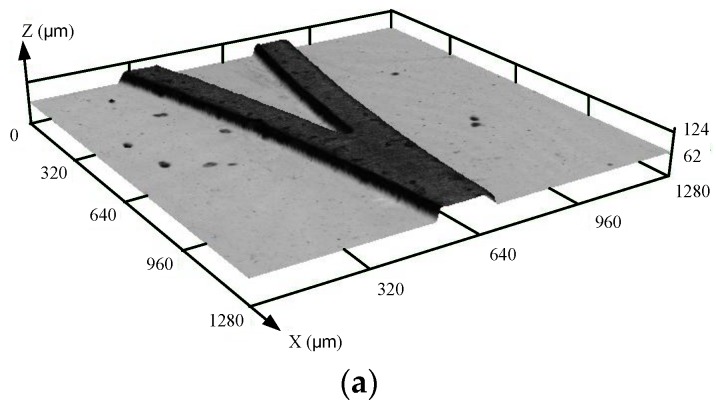

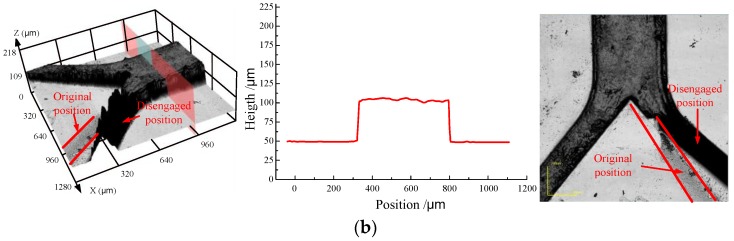
Comparison of the metal micro molds before and after the pull force test. (**a**) Metal micro mold before pull force test; (**b**) Metal micro mold after pull force test.

**Figure 13:**
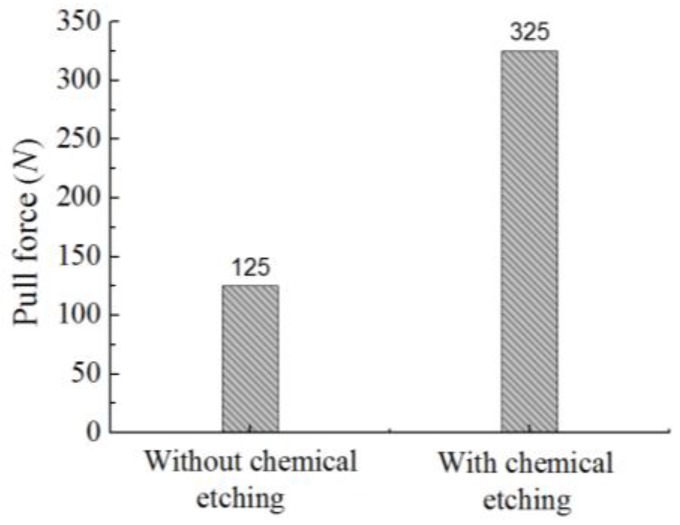
Comparison of pull forces.

In this test, the pull force was increased by 5 N each time until the micro mold was separated from the substrate. Before the tensile test, the metal micro mold was well joined with the substrate (Figure 12a), and during the test, there was separation from the substrate (Figure 12b). The values of the pull force for the micro mold separated from substrate are shown in Figure 13. It can be seen that the pull force was about 125 N when the metal micro mold was separated from the substrate without chemical etching pretreatment, whereas, with the chemical etching pretreatment, the micro mold and substrate were well joined until the pull force increased to 325 N, and the adhesion strength was about 2.6 times than that without chemical etching pretreatment. The chemical etching was a useful way to enhance the bonding strength between the metal micro mold and substrate.

### 3.3. Parameters Optimization for Improving the Surface Roughness of Metal Micro Molds

In Section 3.1 and Section 3.2, it has been proved that additive and chemical etching pretreatment are useful to avoid the pits on the deposited surface as well as enhance the bonding strength between the metal micro mold and substrate. For the metal micro mold, the surface roughness is also a main index, as it directly affects the demold process and the surface quality of the micro product. During the pulse electroforming process, pulse frequency, pulse duty cycle, current density and temperature have significant effect on the surface roughness of the metal mold. In this section, an orthogonal experiment was designed to optimize the parameters. The electroforming process parameters with different levels are listed in Table 2, and all the experiments were performed with chemical etching pretreatment and additive in the solution.

**Figure 14:**
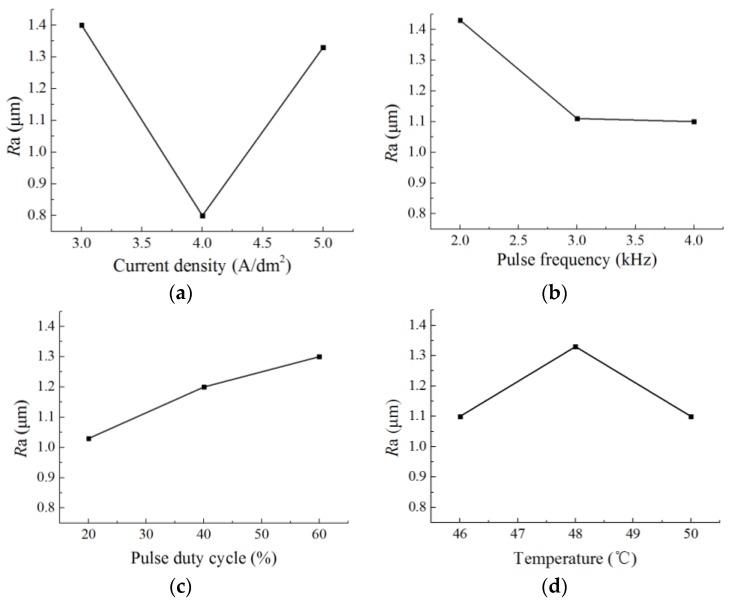
The influence of different factors on the surface roughness of a metal micro mold. (**a**) Current density; (**b**) Pulse frequency; (**c**) Pulse duty cycle; (**d**) Temperature.

The responses of each parameter to surface roughness are shown in in Table 3 and Figure 14. It can be seen that current density was the primary factor that influences the surface roughness, and pulse frequency was the secondary factor. Compared with current density and pulse frequency, the pulse duty cycle and temperature showed no apparent influence on the surface roughness.

Figure 14a shows the influence of current density on surface roughness; it can be seen that the surface roughness of the nickel deposit under current densities of 3 A/dm^2^, 4 A/dm^2^ and 5 A/dm^2^ were approximately 1.4 μm, 0.8 μm and 1.33 μm, respectively. Also the lowest surface roughness was at the current density of 4 A/dm^2^. The change in surface roughness can be attributed to the increase in cathode polarization and throwing power that is caused by the increasing current density. High cathode polarization promoted the generation of new nuclei so that the grains were refined, which reduced the surface roughness with the current density increasing from 3 A/dm^2^ to 4 A/dm^2^. While the concentration polarization was aggravated with the current density further increased to 5 A/dm^2^, it not only promoted a hydrogen evolution reaction but also inhibited the generation of new nuclei [19,20]. Additionally, the grains became coarse, leading to a high surface roughness. Figure 14b,c show that the surface roughness decreased with the increasing pulse frequency but increased with the increasing pulse duty cycle. This can be explained that in pulse current, a low pulse width reduced the growing time of new nuclei, which was useful for refining the grains. Reducing the pulse duty cycle and increasing pulse frequency meant a low pulse width, and then the grains were refined, which promoted a low surface roughness. In addition, Figure 14d shows that the influence of temperature on the surface roughness was not evident compared with other factors, and the change was about 0.23 μm with the increasing temperature.

**Figure 15:**
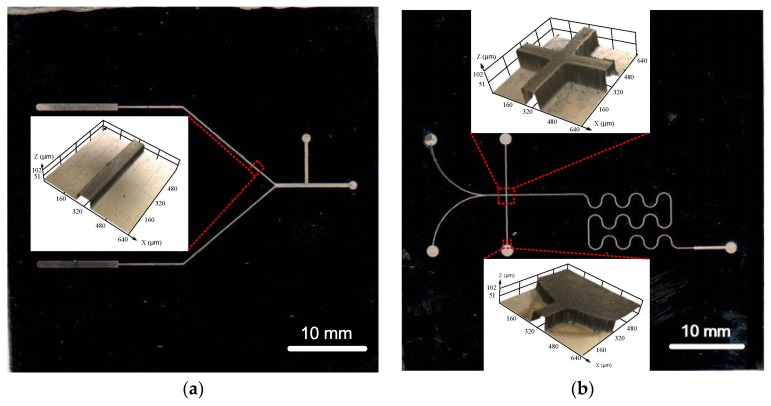
Metal micro molds prepared with different structures.

According to the results of the orthogonal experiment, A_1_, B_3_, C_2_ and D_3_ could be used as the optimized parameters for electroforming the metal micro mold, that is, a pulse frequency of 4 kHz, pulse duty cycle of 20%, current density of 4 A/dm^2^ and a temperature of 46 °C. By using the optimized parameters, metal micro molds with different structures were well prepared with a minimum roughness of 0.56 μm, as shown in Figure 15. The images of metal micro molds were generated by using a scanner (HP LJ M225 Scan, Hewlett-Packard Company, Palo Alto, CA, USA).

### 3.4. Hot Embossing Experiment with the Micro Metal Mold

In order to investigate the quality of the micro metal mold, a preliminary hot embossing experiment was performed, and the actual image of the micro metal mold was used as shown in Figure 15a.

In the hot embossing experiment, the PMMA material was employed, and the experiment was performed by using a compact nano-imprint tool (CNI v2.0, Beijing E-Science Co., Ltd., Beijing, China). The detailed process was as follows:

1. The PMMA substrate with a thickness of 2 mm was placed in the system and heated in vacuum to a temperature of 110 °C, which was above the glass transition temperature (*T*_g_ = 104 °C)
2. The micro metal mold was also heated to the same temperature
3. The micro structure was pressed into the PMMA substrate with a pressure of 0.5 MPa, and the holding time was about 10 min
4. Metal mold and PMMA substrate were isothermally cooled to the temperature of 50 °C and then separated (demolding).

**Figure 16:**
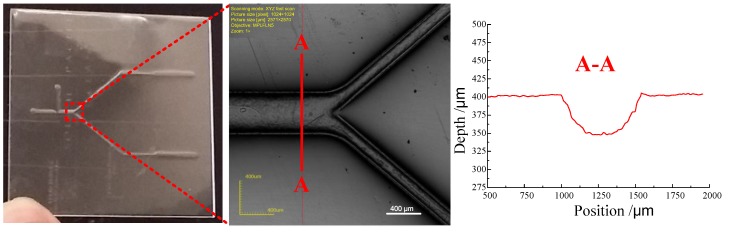
The microfluidic structure prepared with a hot embossing process.

**Figure 17:**
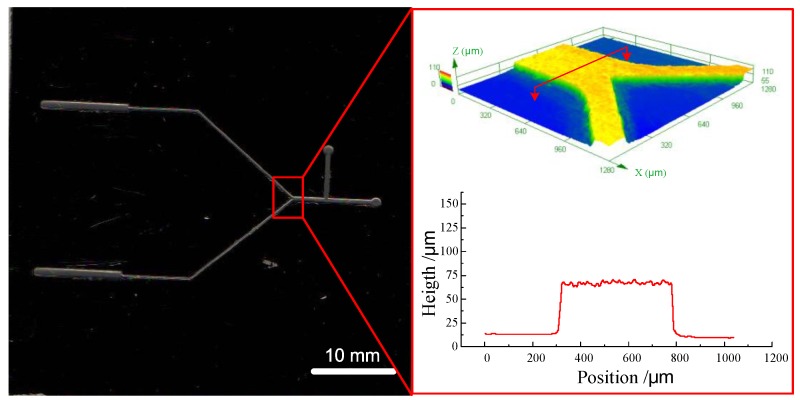
The micro metal mold after the hot embossing experiment.

Figure 16 shows the microfluidic structure prepared with the hot embossing process. After 60 embossing cycles, there was no deposited micro structure falling off the metal substrate (as shown in Figure 17). This indicated that the method used in this paper could well improve the quality of micro metal molds by enhancing the bonding strength between the deposited micro structure and the substrate, which is useful for increasing the service life of the micro metal mold. However, because the hot embossing experiment was preliminary, the parameters were not optimized and the quality of the embossing result was not good. In future work, we will systematically investigate the effect of different embossing parameters on the quality and accuracy of the replica.

## 4. Conclusions

This paper proposed a modified method for preparing metal micro molds by using pulse micro electroforming, and based on the investigation, the following conclusions were obtained:

1. For electroforming micro molds, the additive was beneficial for removing the pits and improving the surface quality of the deposit. NaC_12_H_25_SO_3_ with a concentration of 0.08 g/L was used as the additive.
2. The pretreatment of substrate with chemical etching was helpful for enhancing the bonding strength between the metal micro mold and substrate. The adhesion strength reached 325 N, and it was about 2.6 times stronger than substrate without chemical etching pretreatment.
3. By using orthogonal experiments, the parameters with a pulse frequency of 4 kHz, pulse duty cycle of 20%, current density of 4 A/dm^2^ and a temperature of 46 °C were optimized for electroforming a micro mold with good surface quality. Also, metal micro molds with different structures were well prepared with the minimum roughness of 0.56 μm.
4. The embossing experiment also indicated that by using the method proposed in this paper, there was no deposited micro structure falling off from the metal substrate after the embossing experiment, and the quality of micro metal mold was much improved.

## Figures and Tables

**Table 1:** Experimental parameters.

Composition and Process Condition	Value
Pulse duty cycle	20%, 40%, 60%
Frequency (kHz)	2, 3, 4
Ni(NH_2_·SO_3_)_2_·6H_2_O (g/L)	400
H_3_BO_3_ (g/L)	35
NiCl_2_ (g/L)	15
NaC_12_H_25_SO_3_ (g/L)	0.08
pH	3.8
Temperature (°C)	45

**Table 2:** Electroforming process parameters and levels.

Parameter Factors	Parameter Levels		
	Level 1	Level 2	Level 3
A: Pulse frequency (kHz)	4	3	2
B: Pulse duty cycle (%)	60	40	20
C: Current density (A/dm^2^)	5	4	3
D: Temperature (°C)	50	48	46

**Table 3:** Orthogonal array and result of factor responses to surface roughness.

Exp. No.	A	B	C	D	Surface Roughness (*Ra*/μm)
1	1	1	1	1	0.7
2	1	2	2	2	0.8
3	1	3	3	3	1.5
4	2	3	2	1	1
5	2	1	3	2	1.1
6	2	2	1	3	1.2
7	3	2	3	1	1.6
8	3	1	1	2	2.1
9	3	3	2	3	0.6
*k*_1_	1	1.3	1.33	1.1	-
*k*_2_	1.116	1.2	0.8	1.33	-
*k*_3_	1.43	1.03	1.4	1.1	-
Range	0.43	0.27	0.6	0.23	-
